# Diagnostic Accuracy of Parkinson Disease by Support Vector Machine (SVM) Analysis of ^123^I-FP-CIT Brain SPECT Data: Implications of Putaminal Findings and Age

**DOI:** 10.1097/MD.0000000000000228

**Published:** 2014-12-12

**Authors:** Barbara Palumbo, Mario Luca Fravolini, Tommaso Buresta, Filippo Pompili, Nevio Forini, Pasquale Nigro, Paolo Calabresi, Nicola Tambasco

**Affiliations:** From the Section of Nuclear Medicine and Health Physics, Department of Surgical and Biomedical Sciences (BP, TB, NF); Department of Engineering (MLF, FP); Neurology, Perugia University Hospital and Section of Neurology, Department of Medicine, University of Perugia, Perugia (PN, PC, NT); and I.R.C.C.S. Santa Lucia, Rome, Italy (PC).

## Abstract

Brain single-photon-emission-computerized tomography (SPECT) with ^123^I-ioflupane (^123^I-FP-CIT) is useful to diagnose Parkinson disease (PD). To investigate the diagnostic performance of ^123^I-FP-CIT brain SPECT with semiquantitative analysis by Basal Ganglia V2 software (BasGan), we evaluated semiquantitative data of patients with suspect of PD by a support vector machine classifier (SVM), a powerful supervised classification algorithm.

^123^I-FP-CIT SPECT with BasGan analysis was performed in 90 patients with suspect of PD showing mild symptoms (bradykinesia-rigidity and mild tremor). PD was confirmed in 56 patients, 34 resulted non-PD (essential tremor and drug-induced Parkinsonism). A clinical follow-up of at least 6 months confirmed diagnosis. To investigate BasGan diagnostic performance we trained SVM classification models featuring different descriptors using both a “leave-one-out” and a “five-fold” method. In the first study we used as class descriptors the semiquantitative radiopharmaceutical uptake values in the left (L) and right (R) putamen (P) and in the L and R caudate nucleus (C) for a total of 4 descriptors (CL, CR, PL, PR). In the second study each patient was described only by CL and CR, while in the third by PL and PR descriptors. Age was added as a further descriptor to evaluate its influence in the classification performance.

^123^I-FP-CIT SPECT with BasGan analysis reached a classification performance higher than 73.9% in all the models. Considering the “Leave-one-out” method, PL and PR were better predictors (accuracy of 91% for all patients) than CL and CR descriptors; using PL, PR, CL, and CR diagnostic accuracy was similar to that of PL and PR descriptors in the different groups. Adding age as a further descriptor accuracy improved in all the models. The best results were obtained by using all the 5 descriptors both in PD and non-PD subjects (CR and CL + PR and PL + age = 96.4% and 94.1%, respectively). Similar results were observed for the “five-fold” method.

^123^I-FP-CIT SPECT with BasGan analysis using SVM classifier was able to diagnose PD. Putamen was the most discriminative descriptor for PD and the patient age influenced the classification accuracy.

## INTRODUCTION

One of the most important challenges in Parkinson disease is the differential diagnosis. Diagnostic imaging modalities are useful to ameliorate the clinical suspect. One of the most widely available diagnostic tool is brain SPECT (single-photon-emission-computerized tomography) with ^123^I-ioflupane (^123^I-FP-CIT), a pre-synaptic radiopharmaceutical of the dopaminergic transporters (DAT) presenting a significant uptake decrease in basal ganglia of PD subjects.^1–4^ A semiquantitative analysis^1,4^ of data can be obtained by measuring the radiopharmaceutical activity in basal ganglia, in both caudate nucleus and putamen. Different programs for semiquantitative analysis are currently available^1,4^ and BasGan V2 software is one of the most diffuse.^5^

A recent paper of Nobili et al^6^ investigated the effect of age, gender, handedness, body mass index, time, and season of examination on DAT availability in a group of 122 healthy subjects (aged 18–83 years) evaluated by ^123^I-FP-CIT brain SPECT with BasGan V2 software analysis showing that semi-quantitative values of caudate nuclei and putamina were significantly affected by age and gender. The authors proposed a method to compute if a given subject, clinically confirmed, has likely normal or abnormal values according to the effect of age and gender on caudate nuclei and putamina semi-quantitative values. It was highlighted that the effect of age might be overestimated because BasGan volumetric regions of interest of putamina and caudate nuclei are rigid and do not take into account the possible volume reduction caused by atrophy in the elderly population. In this study, investigating only a selected group of normal subjects, a pattern recognition analysis on different categories of patients was not performed.

In a recent paper Haller et al^7^ proposed a pattern recognition analysis based on brain magnetic resonance (MR) diffusion tensor imaging (DTI) data (multivoxel pattern analysis) to discriminate subjects with PD comparing with other PD-mimicking conditions. They used a support vector machine (SVM) classifier that represents a multivariate tool of “machine learning,” a branch of artificial intelligence. In this study data of 40 consecutive patients with Parkinsonism suggestive of PD with DTI (MR at 3T), brain ^123^I-FP-CIT SPECT and extensive neurologic testing were examined by SVM analysis with the purpose of identifying possible patterns allowing the discrimination of individual subjects.

SVMs implement supervised learning models to analyze data and recognize patterns. SVMs are typically employed for classification and regression analysis. Based on a sets of examples belonging to different diagnostic category, a SVM during the training phase builds a model that can be later used to classify new examples into 1 diagnostic category. The SVM algorithm computes the class separation boundaries with the aim of maximizing the distance between the boundaries and the example points belonging to different classes.^8^ It has been theoretically and empirically shown that SVMs have good generalization capabilities, thus being able to classify also data of new patients (not used in the training phase).^9,10^ A pioneering paper applying SVM in classifying ^123^I-FP-CIT brain SPECT data of patients with Parkinsonian syndromes was published by Prashanth et al^11^ in 2014. The authors investigated data deriving from the Parkinson progression marker initiative (PPMI) database^12^ using as features the striatal binding ratio of the 4 striatal regions (left and right caudate, left and right putamen), showing that SVM was a valuable method to correctly classify PD versus normal subjects.

To contribute to investigate the influence of age in the diagnostic performance of Basal Ganglia V2 software (BasGan),^5,6^ we examined patients with clinical suspect of PD undergoing ^123^I-FP-CIT brain SPECT with BasGan analysis, evaluating results obtained by means of individual-level SVM classification.

## METHODS

^123^I-FP-CIT brain SPECT with semiquantitative analysis with BasGan V2 software^5,6^ was performed in 90 retrospective patients [46 males (M) and 44 females (F), range of age: 37–85 years (yrs)] with mild symptoms (bradykinesia-rigidity and mild tremor) in order to confirm or exclude PD; 56 subjects resulted affected by PD [29 M and 27 F, range of age: 44–85 yrs, Hoehn and Yahr score (HY)^13^: 0.5–1.5; Unified Parkinson Disease Rating Scale (UPDRS)^14^ score: 6–38] and 34 not affected by disease (non-PD, 17 M, 17 F, range of age: 37–82, affected by essential tremor and drug-induced Parkinsonism). A clinical follow-up of at least 6 months confirmed final diagnosis. The study was retrospective and all the patients signed a written informed consent.

**FIGURE 1:**
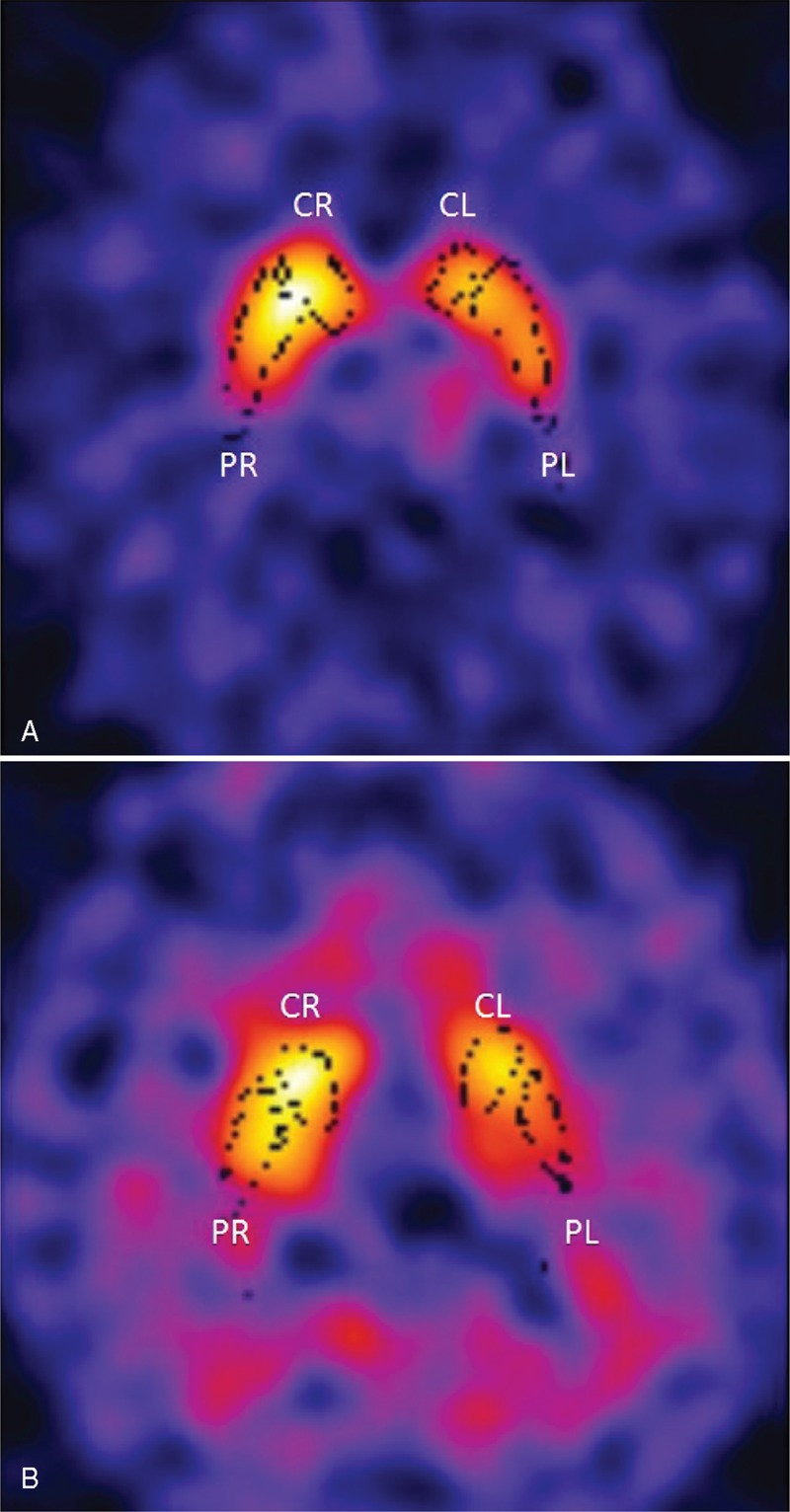
Three-dimensional striatal template positioning provided by BasGan software of the ^123^I-FP-CIT brain SPECT images in the transaxial sections of a non-PD (A) and a PD (B) subject. The 3D template of basal ganglia (black dots) in the SPECT images allows to measure activity in the different striatal regions (CR, CL, PL, PR) and its dimension is fixed. An optimization protocol automatically adjusts the positioning of blurred templates to best match the radioactive counts and places an occipital region of interest (ROI) for background evaluation. The non-PD subject has a normal radiopharmaceutical distribution in putamina and caudate nuclei (A), while the PD patient shows a reduction in radiocompound uptake in putamina (B).

^123^I-FP-CIT (185 MBq, DaTSCAN®, GE Healthcare, Medi-Physics, Inc. Arlington Heights, IL, U.S.A.) was intravenously injected to each patient at least 30 min after potassium perchlorate (400 mg) oral administration to block radiolabelled iodine thyroid uptake. SPECT images were performed 3.5 h after radiopharmaceutical injection by a dual-head gamma camera (Millennium VG; General Electric Medical Systems, Milwaukee, WI, USA), equipped with low-energy high-resolution collimators. Image acquisition parameters were: rotational radius between 13 and 15, 128 × 128 matrix, 120 projections (rotation of 360°C), 40 s/projection; slice thickness was 4.42 mm and image reconstruction was carried out by filtered back-projection with a Butterworth filter (cut-off frequency 0.45, order 10) to produce transaxial slices corrected for attenuation by means of Chang method. Reconstructed images were analyzed to obtain semi-quantification by means of the freely available BasGan V2 software (http://www.aimn.it/struttura/gruppi/gs_neuro.php), based on a highdefinition, 3-D striatal template, derived from Talairach atlas, as previously described.^5,6^ The software allows an automatic, 3-D segmentation of caudate and putamen in each hemisphere. An optimization protocol automatically adjusts the positioning of blurred templates to best match the radioactive counts and places an occipital region of interest (ROI) for background evaluation. Partial volume effect (PVE) correction is performed in the process of binding computation of the caudate nucleus, putamen and background (in occipital cortex) as described^6^ and consists of an activity assignment in a Talairach–Tournoux atlas-based 3-compartment model of basal ganglia. Putamen and caudate nucleus binding was subtracted by background binding as follows [(caudate nucleus or putamen binding − background binding)/background binding] to obtain measurement of the specific to non-displaceable binding ratio (SBR) in caudate nucleus and putamen in each hemisphere. Figure 1 shows the 3-D striatal template positioning in the SPECT transaxial sections of a non-PD and a PD patient of our study.

The semiquantitative data extracted from BasGan in addition to the age of the patients constitute the set of descriptors used to classify patients.

The SVM algorithm available in the matlab programming environment (Matlab Statistics Toolbox, The Mathworks Inc., Natick, MA, USA) was used to train the model with the purpose of predicting the 2 diagnostic categories under investigation. In particular it was implemented a SVM with a Radial Basis Function kernel. To assess the generalization capacity of the SVM classifier a cross-validation statistical analysis was performed by applying both a “leave-one-out” and a “five-fold” method.

To investigate BasGan diagnostic performance and to evaluate the influence of the different descriptors, we trained SVM classification models featuring different subset of descriptors.

In the first study we used as class descriptors the semiquantitative radiopharmaceutical uptake values in the left (L) and right (R) putamen (P) and in the L and R caudate nucleus (C). In the first classification model each patient was described by all the above-mentioned 4 descriptors (CL, CR, PL, PR).

In the second study each patient was described only by the 2 features obtained by CL and CR, while in the third study PL and PR were considered. To evaluate the influence of the age in the classification performance, as additional studies the above 3 studies were repeated by including the age of the patients as a further descriptor. Therefore the overall data set of descriptors available for the classification is the following: CL, CR, PL, PR, age.

## RESULTS

Both cross-validation methods showed that brain SPECT with ^123^I-FP-CIT with BasGan analysis was a valuable tool reaching a correct classification performance higher than 73.9% in all the models. Table 1 reports the overall results for all the SMV models for the 2 cross-validation methods (“leave-one-out” and “five-fold”).

**TABLE 1:**
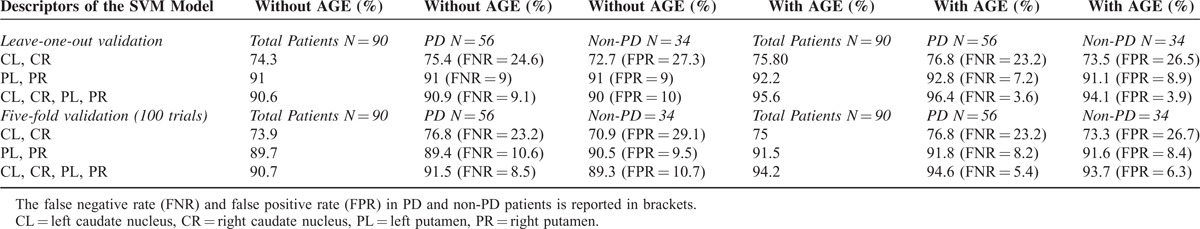
Classification Accuracy of SVM (Support Vector Machine) Analysis (Expressed as Percentual Values, %) With the 3 Models Carried Out With Different Descriptors (CL and CR; PL and PR; CL, CR, PL, and PR) With the 2 Validation Methods (Leave-One-Out and 5-Fold) to Discriminate PD (Parkinson Disease) Patients Versus Non-PD Subjects (Including or Not Age as a Further Descriptor)

Considering the “Leave-one-out” method, putamen descriptors were better predictors (accuracy of 91% for both groups of patients) comparing with caudate nucleus ones, while using values of both putamina and caudate nuclei the diagnostic accuracy was similar to that of putamen only in all the groups of patients.

Adding age as a further descriptor the diagnostic accuracy improved in all the models and the best results were obtained by using all the 5 descriptors in both categories of patients.

Similar results were observed for the “five-fold” cross-validation method, as shown in Table 1. Furthermore no significant difference was evidenced performing classification models separating CL and CR or PL and PR.

## DISCUSSION

Brain SPECT with ^123^I-FP-CIT with BasGan analysis was able to diagnose PD with great accuracy, as evaluated by the automatic classifier SVM. Age played a relevant role because it improved the diagnostic accuracy, thus confirming the data of Nobili et al on normal subjects^6^ and supporting the hypothesis that uptake distribution in the basal ganglia has to be related to the age of the patient to diagnose PD.

Among the different features used to classify patients, putamen resulted as the most discriminative descriptor for Parkinson disease independently of age. Literature is controversial on this topic. Several studies described that DAT tended to decline with patient age in caudate nuclei with respect to putamen,^15–17^ while others reported a DAT reduction with age for both caudate nucleus and putamen.^18,19^ Our data confirm those of Nobili et al^6^ showing that the age-related decline of radiopharmaceutical uptake was more evident in putamen. This can be explained because the BasGan software includes in the 3-D ROIs the caudate body and tail in addition to the head as performed in previous papers. The relatively more sparse nigrocaudate endings in the body-tail comparing with the head might have a role showing a milder DAT decline in caudate nucleus as compared to putamen. On the other hand different results in literature might depend on many factors, such as the radiopharmaceutical used to investigate DAT, the number and the age range of subjects studied and the modalities of ROI drawing. Finally concerning BasGan software Nobili et al remarked that the greater reduction of putamen uptake with aging was unlikely to be an artefact of BasGan 3-D ROI positioning, because their dimension is fixed and not depending on subject age.^6^

Furthermore SVM in our study represented a valuable tool to perform pattern recognition analysis with the purpose of classifying patients. Computer aided diagnosis (CAD) by means of artificial intelligence plays a very promising role in medical imaging as a support to medical diagnosis as many studies demonstrated.^2,7,20–23^ In particular to our knowledge only few studies evaluated ^123^I-FP-CIT brain SPECT data by means of CAD to investigate movement disorders.

Hamilton et al^20^ investigated the differential diagnosis between PD and ET using a neural network (NN) classifier on quantified ^123^I-FP-CIT brain SPECT data, showing that the NN was clearly able to discriminate these pathological categories. For the small number of patients examined in the study (n = 18), in sequence 17 patients were used to test the trained NNs and in each case a new NN was created using randomly generated weights and biases. This process was repeated 18 times in order to consider all the data combinations and a 2-stage analysis was carried out. In the first stage the striatum-to-occipital cortex ratio was assessed to investigate non-early Parkinsonian syndromes (and patients with a low ratio would be stopped at this step), and in the second stage the putamen-to-caudate nucleus ratio was measured. Finally, the 2-stage analysis was undertaken and repeated, in a single step, using an artificial NN. The authors evidenced that the 2-stage analysis was less effective than the single step process using the NN that was clearly able to discriminate between Parkinsonian syndromes and ET in all patients without equivocal results. However, the study had 2 limitations: the “gold standard” outcome represented by the judgement of a single experienced observer and the small and poorly balanced number of patients examined (18 total, 13 with Parkinsonian syndromes, and 5 with ET).

In a subsequent study of our group^2^ examining 216 patients, we used 2 different artificial neural network classifiers, a probabilistic neural network (PNN) and a classification tree (ClT), to evaluate the different diagnostic performance of ^123^I-FP-CIT brain SPECT in the differential diagnosis of PD (in early and advanced phase) and essential tremor (ET). ^123^I-FP-CIT brain SPECT data were analyzed by measuring specific/non-specific putamen/occipital (p/o) and caudate/occipital (c/o) binding ratios of radiopharmaceutical uptake using a standard ROI template manually constructed according to a stereotactic atlas and including fixed regions for both c/o ratio and p/o ratio applied to 3 different representative slices. The 2 classifiers had a similar and relevant capability to distinguish ET patients from the other subjects (96.6 ± 2.6% for PNN, 93.5 ± 3.4% for ClT), while correct classification performances were slightly lower for early PD (81.9 ± 8.1% for PNN, 69.8 ± 5.3% for ClT) comparing with advanced PD (78.9 ± 8.1% for PNN, 88.1 ± 8.8% for ClT). This could be explained because both groups of PD patients belonged to the same clinical category, differing only in disease severity. Comparing the overall results for the classification of patients with early and advanced PD, 1 classifier was not clearly better than the other. However, ClT added further information because it provided reliable cut off values able to differentiate ET and PD of different severities. Patients with putamen values >5.99 were classified as having ET, while patients with putamen values <5.99 were classified as having PD. Furthermore, if the caudate nucleus value was >6.97 patients were classified as early PD (probability 69.8 ± 5.3%), while if the value was <6.97 patients were classified as advanced PD (probability 88.1 ± 8.8%). Although this work included a relevant number of patients (216 subjects examined, being 89 affected by ET, 64 by early PD, and 63 by advanced PD), it was carried out by a semiquantitative ROI analysis that has the limit to consider manually selected not volumetric ROIs. In this present study we performed semiquantitative analysis of brain SPECT data by BasGan software, a volumetric 3D ROI method of putamina and caudate nuclei, further evaluating the influence of age in the data obtained.

Haller et al^7^ performed a pattern recognition analysis by SVM to diagnose subjects with PD at the individual level, basing only on DTI data, while ^123^I-FP-CIT brain SPECT was a reference diagnostic tool requested in the inclusion criteria (when suggestive for PD or Parkinsonism) but not considered for SVM analysis.

As previously affirmed by Haller et al^7^ in the study on DTI in PD, it is worth of mention that the individual-level SVM classification is different from the generally performed group-level voxelwise analysis that represents univariate test that separately analyzes each included voxel between different categories. SVM analysis is a multivariate tool able to identify patterns allowing the discrimination of individual subjects. There is only 1 resulting parameter per subject and therefore no corrections for multiple comparisons are needed. This contributes to obtain a better diagnostic classification of our patients.

Finally the paper of Prashanth et al^11^ assessed patients undergoing ^123^I-FP-CIT brain SPECT for the early diagnosis of PD by using SVM and logistic regression in the model building process, showing that both methods had a high accuracy in discriminating early PD and healthy subjects. The strengths of this interesting study are the very high sample of patients examined (369 early PD and 179 normal subjects) deriving from the above mentioned multi-centre database^12^ and the high classification performance reached using only 4 features (left and right caudate, left and right putamen) avoiding any feature selection techniques and thus making the system more robust. Furthermore the authors compared the diagnostic performance of SVM carried out by RBF and linear kernel functions showing that RBF kernel provided a higher classification accuracy than linear kernels and other methods available in literature.

We also implemented different architectures for SVM using different kernel functions, observing in preliminary tests that linear kernel provided worse performances than RBF kernels (2–3% in classification accuracy). For this reason we employed RBF kernel in this study, as suggested by previous data.^11^

Therefore our study is in line with the paper of Prashanth, supporting the ability of SVM analysis with RBF kernel to discriminate PD patients undergoing ^123^I-FP-CIT brain SPECT, but it includes also the influence of age as a further descriptor in the model. In the paper of Nobili et al^6^ both age and gender were the most significant factors influencing the specific to non-displaceable binding ratios in the normal subjects. Our retrospectively studied patients were matched for gender differences, so only the influence of age was investigated.

Finally a limit of this study is the unavailability of healthy control subjects, because we examined patients for clinical purposes having mild symptoms (bradykinesia-rigidity and mild tremor) to confirm or exclude PD. Therefore our patients are considered as having PD or not (non-PD group), but we do not have a further normal group of subjects.

In conclusion in our study we evaluated the diagnostic performance of ^123^I-FP-CIT brain SPECT in identifying PD subjects by SVM analysis, showing that putamen was the most discriminative descriptor for PD and that the patient age was able to influence the classification accuracy.
